# The Effects of an Energy Drink on Psychomotor Vigilance in Trained Individuals

**DOI:** 10.3390/jfmk4030047

**Published:** 2019-07-22

**Authors:** Jose Antonio, Madaline Kenyon, Christopher Horn, Lia Jiannine, Cassandra Carson, Anya Ellerbroek, Justin Roberts, Corey Peacock, Jaime Tartar

**Affiliations:** 1 Exercise and Sport Science, NSU Florida, Davie, FL 33328, USA; 2 School of Psychology and Sport Science, Anglia Ruskin University, East Road, Cambridge CB5 8DZ, UK; 3 Psychology and Neuroscience, NSU Florida, Davie, FL 33328, USA

**Keywords:** caffeine, exercise, attention, focus, performance

## Abstract

The psychomotor vigilance test (PVT) measures one’s behavioral alertness. It is a visual test that involves measuring the speed at which a person reacts to visual stimuli over a fixed time frame (e.g., 5 min). The purpose of this study was to assess the effects of an energy drink on psychomotor vigilance as well as a simple measure of muscular endurance (i.e., push-ups). A total of 20 exercise-trained men (*n* = 11) and women (*n* = 9) (mean ± SD: age 32 ± 7 years; height 169 ± 10 cm; weight; 74.5 ± 14.5 kg; percent body fat 20.3 ± 6.2%; years of training 14 ± 9; daily caffeine intake 463 ± 510 mg) volunteered for this randomized, double-blind, placebo-controlled, crossover trial. In a randomized counterbalanced order, they consumed either the energy drink (ED) (product: BANG^®^, Weston Florida) or a similar tasting placebo drink (PL). In the second visit after a 1-week washout period, they consumed the alternate drink. A full 30 min post-consumption, they performed the following tests in this order: a 5-min psychomotor vigilance test, three sets of push-ups, followed once more by a 5-min psychomotor vigilance test. Reaction time was recorded. For the psychomotor vigilance test, lapses, false starts and efficiency score are also assessed. There were no differences between groups for the number of push-ups that were performed or the number of false starts during the psychomotor vigilance test. However, the ED treatment resulted in a significantly lower (i.e., faster) psychomotor vigilance mean reaction time compared to the PL (*p* = 0.0220) (ED 473.8 ± 42.0 milliseconds, PL 482.4 ± 54.0 milliseconds). There was a trend for the ED to lower the number of lapses (i.e., reaction time > 500 milliseconds) (*p* = 0.0608). The acute consumption of a commercially available ED produced a significant improvement in psychomotor vigilance in exercise-trained men and women.

## 1. Introduction

Several investigations have assessed the potential ergogenic effects of energy drinks with some showing benefits and others showing neutral effects [1,2,3,4,5,6,7,8,9,10,11]. In a study of six male and six female trained cyclists, consuming Red Bull Energy Drink, they improved performance during a 1-hour cycling time trial. However, there was no effect on the rating of perceived exertion [12]. An energy drink that contained a dose equal to three mg of caffeine per kilogram of body weight increased repeated sprint performance, jump height, as well as performance during a simulated soccer game [5]. On the other hand, an investigation of 15 collegiate soccer players demonstrated a null effect. Mean sprint time was similar between Red Bull (11.31 s) and placebo (11.35 s). In addition, there was no effect of Red Bull consumption on ratings of perceived exertion [6]. Other investigators have shown that there is a significant increase in energy expenditure in young, healthy women after consuming an energy drink; in addition, an increased time to exhaustion during an endurance run with improved subjective measures of focus, energy and fatigue [11,13]. However, to date there are no studies that have examined the effects of caffeine-containing energy drinks on psychomotor vigilance. Thus, the purpose of this investigation was to assess the effects of an energy drink (BANG^®^) on psychomotor vigilance as well as push-up performance. It was hypothesized that improvements in both psychomotor vigilance and push-up performance would result from energy drink consumption when compared to a placebo.

## 2. Materials and Methods 

### 2.1. Participants

A total of 20 exercise-trained men (*n* = 11) and women (*n* = 9) volunteered for this randomized, double-blind, placebo-controlled, counterbalanced, crossover trial. Subjects came to the laboratory on two occasions for performance testing. In accordance with the Helsinki Declaration, the University’s Institutional Review Board approved all procedures that involved human subjects (IRB#: 2019-62-NSU). Written informed consent was obtained prior to participation. Caffeine intake and exercise history was ascertained via a simple questionnaire. The questionnaire asked each subject for the number of caffeine-containing drinks they typically consumed on a daily basis as well as the type of drink (e.g., coffee, tea, colas, energy drinks, etc.). Regarding exercise history, subjects provided data in terms of the number of hours per week that they performed aerobic and resistance training exercises.

### 2.2. Body Composition

Height was assessed prior to body composition assessment via a stadiometer. Body composition (i.e., weight, fat mass, lean body mass, percentage fat) was assessed with a multi-frequency bioelectrical impedance device (InBody 770) to determine each subject’s physical characteristics. Subjects were instructed to arrive at the laboratory after a 3 h fast. Study participants stood on the platform of the device barefoot with the soles of their feet on the electrodes. They then grasped the handles of the unit with their thumb and fingers to maintain direct contact with the electrodes. They stood still for ~1 min while maintaining their elbows fully extended and their shoulder joint abducted to about a 30 degree angle. Body composition was assessed at baseline only.

### 2.3. Energy Drink

**Figure 1:**
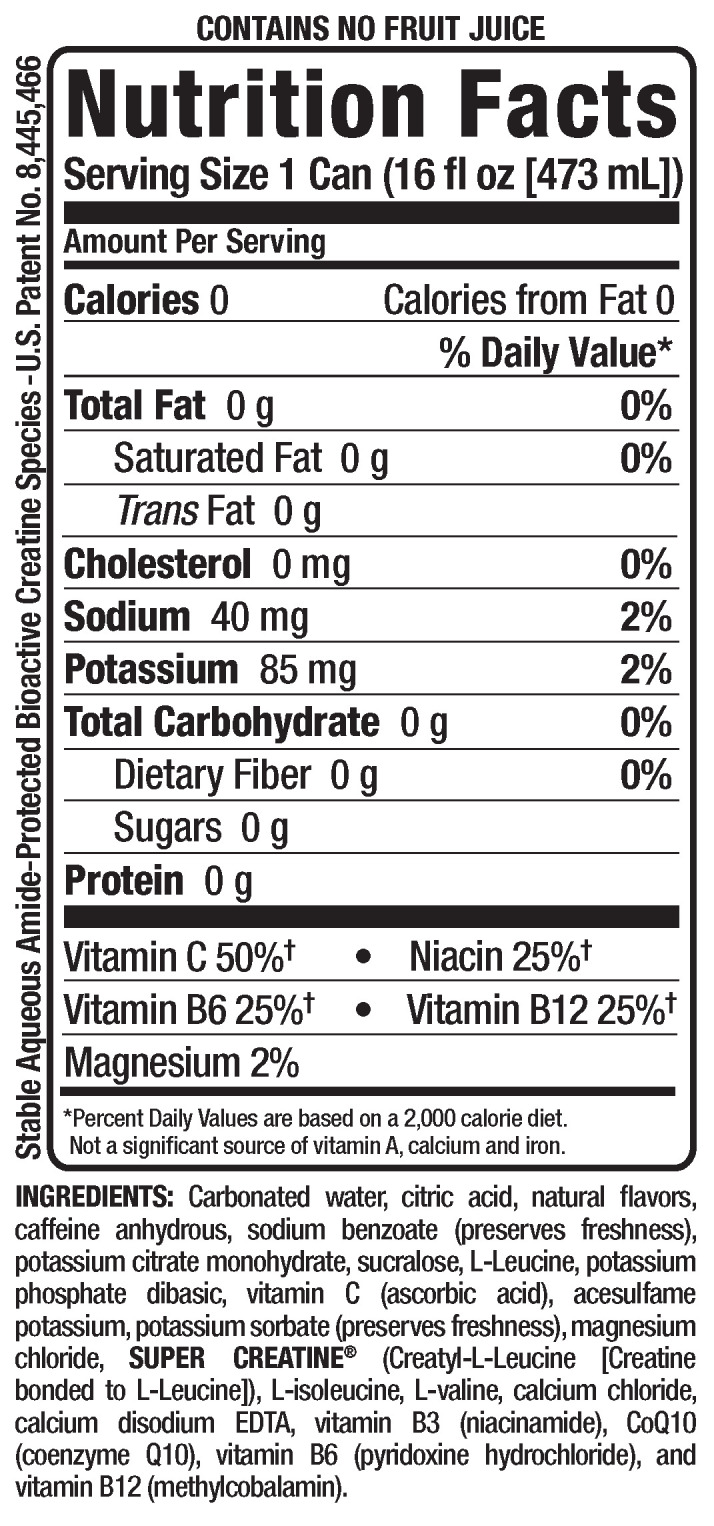
Nutrition Facts panel of BANG energy drink.

The energy drink (BANG^®^) and placebo were provided as a donation from VPX (Weston, FL USA) (Figure 1). The primary active ingredient in the energy drink is caffeine (300 mg per one 473 mL can). The placebo contained the same ingredients and was flavored similarly; however, the placebo did not contain caffeine, creatine, or branched-chain amino acids. The cans of the ED as well as PL were identical. 

### 2.4. Psychomotor Vigilance Task (PVT)

PVT testing was conducted using an automated tests (Joggle Research, Seattle, WA, USA) on a standard electronic tablet (Apple iPad). The PVT requires participants to quickly respond to the sudden appearance of stimuli inside a box and avoid responding prematurely [14]. The PVT measures vigilant attention and activates the prefrontal cortex, motor cortex, and visual cortex [15].

### 2.5. Performance Testing Procedures

Subjects came to the lab after a 3 h fast on two occasions separated by one week between 1100 and 1400 h. They consumed either the energy drink or placebo. A full 30 min post-consumption, they performed a 5-min psychomotor vigilance test (PVT) followed by three sets of push-ups and then repeated the 5-min PVT. For push-ups, each subject was instructed to do the maximal number of push-ups in one minute for three sets. After the first and second set, subjects were allowed a 1-min rest interval. The subject was asked to start in the “up” position (i.e., elbows fully extended, torso straight). The participant’s hands were pointing forward and under the shoulder, with the torso in an extended position. The subject then lowered his or her body by flexing the elbow joint and then returning to the “up” phase. The participant was asked to not let his or her torso touch the mat. The maximal number of push-ups performed consecutively during each 1-min push-up test was recorded. The total number of push-ups from the three sets was used for data analysis. The psychomotor vigilance test is used to assess sustained-attention and reaction time. Participants are to quickly respond to the sudden appearance of stimuli inside a box (i.e., numbers will appear at random times) by tapping the box as quickly as possible (i.e., reaction time) and avoid responding prematurely (i.e., false starts). This test lasted five minutes and was performed twice. There was a 7-day washout period between tests. Subjects returned to the lab the following week, consumed the alternate drink, and repeated the same tasks.

### 2.6. Statistical Analysis

All data is presented as the mean ± SD. GraphPad (Prism 6) software was used for statistical analyses. A paired t-test was used to determine if statistically significant differences (*p* < 0.05) existed between the placebo and treatment. Cohen’s d was used to assess the appropriate effect size. A Kolmogorov–Smirnov test was used to assess normality. The scores from the two 5-min psychomotor vigilance tests were combined for the analysis.

## 3. Results

The physical characteristics of the study participants were as follows: 20 exercise-trained men (*n* = 11) and women (*n* = 9) (mean ± SD: age 32 ± 7 years; height 169 ± 10 cm; weight; 74.5 ± 14.5 kg; percent body fat 20.3 ± 6.2%; years of training 14 ± 9; hours per week of aerobic training 5.0 ± 4.9; hours per week of resistance training 6.5 ± 3.4; mean daily caffeine intake 463 ± 510 mg). 

**Figure 2:**
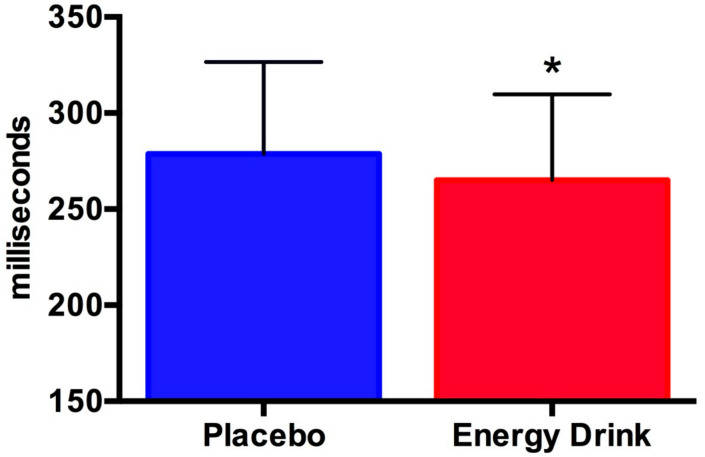
Psychomotor vigilance. Data are shown as the mean ± SD. The energy drink treatment improved PVT reaction time (* *p* = 0.0220).

**Figure 3:**
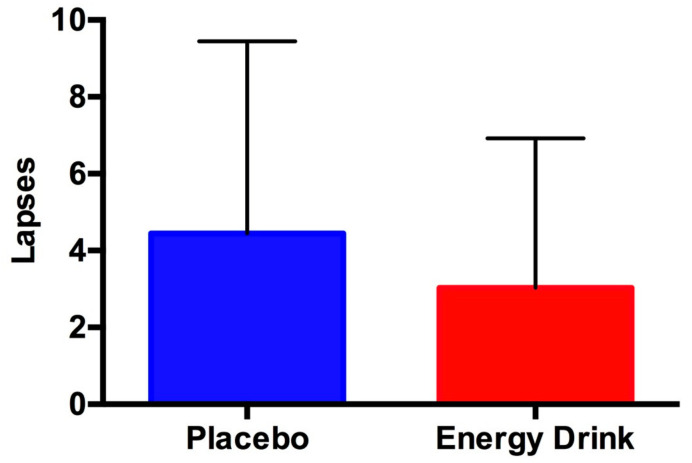
Lapses. Data are shown as the mean ± SD. A lapse is defined as a reaction time > 500 milliseconds. The energy drink treatment also had fewer lapses during the PVT (*p* = 0.0608).

The scores from the first and second psychomotor vigilance tests were combined (Table 1, Figure 2 and Figure 3). Statistical significance was found for one parameter (i.e., psychomotor vigilance) (Table 1). The effect size of this parameter was 0.3. All data demonstrated a normal distribution.

## 4. Discussion

As hypothesized, the current investigation found that the acute consumption of a caffeine-containing energy drink (ED) (i.e., BANG energy drink) significantly improved reaction times of the PVT test in exercise-trained men and women; however, the investigation found no effect on muscular endurance (i.e., push-up performance). The International Society of Sports Nutrition position paper on Energy Drinks stated that although energy drinks contain a number of nutrients that are purported to enhance performance, the primary ergogenic aid is either carbohydrate or caffeine [16]. In the current investigation, the treatment did not contain carbohydrates; thus, the focus of this discussion will be regarding the effects of caffeine.

Magrini et al. assessed the effects of a commercially available energy drink on exercise performance in 31 healthy men and women. They found that both the energy drink and placebo group significantly improved total push-ups and push-ups completed in each of the three sets when compared to baseline. However, there was no difference between the energy drink versus the placebo group [1]. A total of 17 resistance-trained participants consumed placebo or six mg of caffeine per kilogram of body weight one hour before performance testing. Muscular power was assessed with seated medicine ball throw and vertical jump exercises, muscular strength with one-repetition maximum (1RM) barbell back squat and bench press exercises, and muscular endurance with repetitions of back squat and bench press exercises (load corresponding to 60% of 1RM) to momentary muscular failure. Rating of perceived exertion and peak power were assessed immediately after the completion of the back squat and bench press exercises. Compared to placebo, caffeine intake enhanced one-repetition maximum back squat and seated medicine ball throw performance; however, they found no effects on muscular endurance [17]. Chtourou et al. examined the relationships between the effects of consuming a caffeine-containing energy drink (i.e., Red Bull) upon reaction time as well as other performance parameters [18]. In an identical study design as the current investigation (i.e., randomized, double-blind, placebo-controlled, counterbalanced crossover), these investigators found that the acute consumption of the energy drink decreased reaction time, and improved short-term maximal performance (i.e., Wingate test and handgrip strength). 

In an investigation by Beck et al. they discovered an increase in bench press strength (one-repetition maximum) yet there was no effect on muscular endurance measured as total volume of weight lifted during an endurance test with 80% of the one-repetition maximum [19]. In agreement with these studies, the current investigation did not find an effect of energy drink consumption on muscular endurance as measured via the maximal number of push-ups. On the other hand, Stein et al. found that in a small sample of CrossFit trained men, consuming caffeine at a dose of five mg per kilogram of body weight 60 min prior to exercise resulted in an ergogenic effect. Study participants completed as many rounds as possible in 20 min of five pull-ups, 10 push-ups, and 15 air squats. Performance was determined via the total number of repetitions completed in 20 min [20]. One might speculate that the caffeine will likely induce an ergogenic effect regarding muscular endurance if the task is prolonged. In our investigation, three sets of push-ups likely presented an exercise challenge that was of insufficient duration; particularly since our subjects had a substantial number of years in exercise training history. 

The effect of caffeine on the central nervous system or CNS is primarily via its antagonism of adenosine receptors [21]. As little as 32 mg of caffeine can improve auditory vigilance and visual reaction time [22]. Our investigation discovered that the acute consumption of an energy drink improved performance in the psychomotor vigilance task or PVT. What is interesting is that our study participants were not sleep-deprived during the time of the assessment. For instance, McLellan et al. assessed the effects of caffeine on vigilance, marksmanship, and run performance during 27 h of sustained wakefulness in Special Forces personnel. Indeed they found that caffeine maintained vigilance and improved running performance during an overnight field operation in these soldiers [23]. Another investigation found that in healthy adult subjects, the acute consumption of 60 mg of caffeine improved sustained attention and alertness [24]. It should be noted that the dose of caffeine in the current study was 300 mg or approximately four mg of caffeine per kilogram of body weight. It is known that doses of caffeine in the range of three to six mg per kg body weight are effective for enhancing exercise performance [25]. An improvement in PVT would be of benefit for sports that require sustained attention (e.g., baseball, softball, race car driving, etc.). Despite the rather small effect size (0.3), it should be noted that differences in athletic competition are also rather small. That is, all one needs is a small effect from an intervention to result in athletic success.

Limitations of this study include a relatively small sample of trained men and women; thus, it would not be possible to discern if sex differences were present. Furthermore, the role of genotype and how it affects caffeine metabolism was not assessed in this investigation. Nevertheless, our data does agree with prior work vis a vis enhanced performance.

## 5. Conclusions

The acute consumption of an energy drink at a dose of approximately four mg of caffeine per kilogram of body weight significantly improved psychomotor vigilance performance, which is a sustained attention task. It would be interesting to determine what the minimally effective dose would be for a group of exercise-trained individuals.

## Figures and Tables

**Table 1:** Performance Data.

	Placebo	Energy Drink	*p* Value
Psychomotor vigilance (msec)	278.8 ± 47.7	265.0 ± 44.7	0.0220
Number of lapses (>500 msec RT) for the PVT	4.5 ± 5.0	3.0 ± 3.9	0.0608
False starts (total number) for the PVT	1.7 ± 3.3	1.3 ± 3.3	0.3056
Push-ups (number of repetitions)	91.9 ± 35.7	89.4 ± 38.2	0.4324

Data are expressed as the mean ± SD. Legend: msec—millisecond; PVT—psychomotor vigilance test; RT—reaction time.
